# Repurposing FDA-approved drugs as therapeutics to treat Rift Valley fever virus infection

**DOI:** 10.3389/fmicb.2015.00676

**Published:** 2015-07-08

**Authors:** Ashwini Benedict, Neha Bansal, Svetlana Senina, Idris Hooper, Lindsay Lundberg, Cynthia de la Fuente, Aarthi Narayanan, Bradford Gutting, Kylene Kehn-Hall

**Affiliations:** ^1^National Center for Biodefense and Infectious Diseases, School of Systems Biology, George Mason UniversityManassas, VA, USA; ^2^Chemical, Biological, Radiological Defense Division, Naval Surface Warfare CenterDahlgren, VA, USA

**Keywords:** rift valley fever virus, Raf, sorafenib, viral egress, replication, FDA

## Abstract

There are currently no FDA-approved therapeutics available to treat Rift Valley fever virus (RVFV) infection. In an effort to repurpose drugs for RVFV treatment, a library of FDA-approved drugs was screened to determine their ability to inhibit RVFV. Several drugs from varying compound classes, including inhibitors of growth factor receptors, microtubule assembly/disassembly, and DNA synthesis, were found to reduce RVFV replication. The hepatocellular and renal cell carcinoma drug, sorafenib, was the most effective inhibitor, being non-toxic and demonstrating inhibition of RVFV in a cell-type and virus strain independent manner. Mechanism of action studies indicated that sorafenib targets at least two stages in the virus infectious cycle, RNA synthesis and viral egress. Computational modeling studies also support this conclusion. siRNA knockdown of Raf proteins indicated that non-classical targets of sorafenib are likely important for the replication of RVFV.

## Introduction

Rift Valley fever virus (RVFV) is the causative agent of Rift Valley fever, a zoonotic arthropod-borne emerging infectious disease endemic to sub-Saharan Africa that has recently spread to parts of the Arabian Peninsula (Ikegami and Makino, 2011). There is concern that the disease may be able to spread out of endemic areas and further into Asia and Europe or even as far as the western hemisphere (Ikegami, 2012; Rolin et al., 2013). In the majority of human cases, patients are either asymptomatic or experience a mild self-limiting febrile illness. However, some cases can be potentially fatal with symptoms ranging from hemorrhagic fever, hepatitis, meningoencephalitis or ocular damage (Ikegami and Makino, 2011). Besides mosquito bite, humans can be exposed to the virus via the inhalational route through contact with infected animal bodily fluids or with contaminated carcasses post-mortem. Aerosol exposure is a route of concern for use of RVFV as a bioweapon (Caroline et al., 2014).

RVFV affects domestic ruminants with severe economic consequences. Pregnant livestock such as goats, sheep, camels and cattle that contract RVFV experience high rates of spontaneous abortion. In addition, younger animals that become infected have very high mortality rates (Nicholas et al., 2014). Due to the potential to be vectored by mosquito species endemic to the United States (US) (Golnar et al., 2014), importation of viremic livestock (Rolin et al., 2013), the possibility of its weaponization, the devastating possible economic burden and morbidity rates, RVFV has been classified as a Category A pathogen and an overlap select agent by the National Institutes of Allergy and Infectious Diseases (NIAID) and the US Department of Agriculture (USDA). There are currently no FDA-approved vaccines or therapeutics to prevent or treat RVFV infection in humans or ruminants (Caroline et al., 2014). Therefore, more research must be done to develop safe and effective therapeutics to combat this virus.

To address this need, a library of FDA-approved drugs was screened for efficacy of RVFV inhibition. Candidates were evaluated based on the magnitude of viral inhibition and cellular toxicity. Several clusters of drugs with similar pathway or protein targets were identified including inhibitors of growth factor receptors, microtubule assembly/disassembly, and DNA synthesis. One candidate that was able to inhibit virus levels to the greatest extent with no toxicity was sorafenib. Sorafenib blocks the autophosphorylation of a number of receptor tyrosine kinases (RTKs) and inhibits downstream Raf kinases (such as B- and C-Raf, Roberts and Der, 2007). To further validate the efficacy of sorafenib against RVFV infection we examined sorafenib effects in both *in vitro* and *in vivo* models. We observed that sorafenib could decrease RVFV replication by several logs and increased the survival of mice infected with virulent RVFV strain, ZH501. Finally, experiments to delineate at what point of the virus lifecycle sorafenib was affecting and possible mechanism of inhibition were performed.

## Materials and methods

### Cell culture

Vero (ATCC CCL-81) and 293T (ATCC CRL-3216) cells were grown in Dulbecco's modified minimum essential medium (DMEM) supplemented with 10% heat-inactivated fetal bovine serum (FBS), 1% penicillin/streptomycin and 1% L-glutamine. Human small airway epithelial cells (HSAECs) (Popova et al., 2010) were grown in Ham's F12 containing 10% FBS, 1% penicillin/streptomycin, 1% L-glutamine, 1% non-essential amino acids (NEAA), 1% sodium pyruvate and 0.1% 1000X beta-mercaptoethanol (Invitrogen). Huh7 cells were grown in DMEM containing 1% L-glutamine, 1% NEAA, 10% FBS, 1% penicillin/streptomycin and 1% sodium pyruvate. BHK-J cells, a BHK-21 derivative (Lindenbach and Rice, 1997) were maintained in MEM media containing 1% L-glutamine, 1% penicillin/streptomycin, and 7.5% FBS. BSR-T7/5 cells, a BHK-21 cell clone stably expressing T7 RNA polymerase (Buchholz et al., 1999), were cultured similarly as BHK-J cells with the addition of 500 μg/mL geneticin. All cell lines were maintained at 37°C in humidified 5% CO2. Unless noted otherwise, all cells were plated at a density of 5.0 × 10^5^ cells cultured in 6-well plates, 2.5 × 10^5^ cells cultured in 12-well plates, and 1 × 10^4^ cells cultured in 96-well plates.

### Viruses

Recombinant (r)MP12 virus was rescued by transfection of BSR-T7/5 cells with the following plasmids: pProT7-M(+), pProT7-L(+), pProT7-S(+), pT7-IRES-vN, pT7-IRES-vL, and pCAGGS-vG (Ikegami et al., 2006; Kalveram et al., 2011). To generate an initial seed stock, cells (seeded at 3 × 10^6^ cells per 75 cm^2^ flask) were transfected with 4 μg each of pProT7-M(+), pProT7-L(+), pProT7-S(+), pT7-IRES-vN and 2 μg each of pT7-IRES-vL, and pCAGGS-vG using TransIT-LT1 (Mirus). Ratio of total plasmid DNA amount (μg) to TransIT-LT1 volume (μL) was kept at 1:3. Complete media without geneticin selection was used during transfection and subsequent culturing. At 24 h post transfection, media was removed, cells washed once, and complete media added back. After an additional 72 h, media supernatants were collected, clarified by centrifugation (5 min, 3000 rpm, 4°C), aliquoted, and stored at -80°C. Infectious viral titers were determined by plaque assay on Vero cells.

To generate the seed stock of rZH548 virus, co-cultures of 293T and BHK-J cells (1:1 ratio, 3.0 × 10^5^ cells/well) were transfected with the following plasmids: pHH21-RVFV-vL, pHH21-RVFV-vM, pHH21-RVFV-vS, pI.18-RVFV-L, and pI.18-RVFV-N (Habjan et al., 2008). As described above, a 6-well plate was transfected using TransIT-LT1 reagent combined with 4 μg plasmid DNA mixture (1 μg each of the viral RNA plasmids and 0.5 μg each of the viral protein-encoding plasmids) per well. Media supernatants for individual wells were collected and viral titers determined by plaque assay on Vero cells.

To generate a P1 viral stock, subconfluent monolayers of Vero cells were infected at a multiplicity of infection (MOI) 0.1 for 1 h. Inoculum was then removed, cells washed once, and complete media added. Two days later when cytopathic effect was observed within the culture, media supernatants were harvested twice and stored at 4°C. After the last collection, supernatants were then pooled together, filtered (0.2 μM), and stored at −80°C in aliquots. Viral titers were determined by plaque assay on Vero cells.

RVFV ZH501 was obtained from Stuart Nichol, Centers for Disease Control and Prevention. Upon receipt, the virus was passaged once in Vero cells and sucrose purified prior to use in mouse experiments.

### FDA-approved drug libraries and treatment

A library of FDA-approved drugs was purchased from Selleckchem (# L1300) and used for *in vitro* studies. Drugs were received resuspended in DMSO at 10 mM. The drugs were further diluted to a concentration of 10 μM in culture media for use in *in vitro* experiments. Sorafenib tosylate used for *in vitro* studies was also purchased from Selleckchem (# S1040) while sorafenib tosylate used for *in vivo* studies was purchased from Eton Bioscience Inc. (# 1100205002). Curcumin (Sigma) was used at 10 μM. For all inhibitor treatments, cells were pretreated for 1 h before RVFV infection. After viral inoculum was removed and cells washed, new media containing inhibitor was placed back on cells. Unless noted otherwise, cells were cultured for an additional 24 h. As a control, DMSO alone was included for comparison.

### Luciferase assays

Cells plated in a 96-well plate were infected with RVFV MP12 ΔNSs-Luc (MP12 lacking the NSs gene and replaced by a gene encoding *Renilla* luciferase) at a MOI 0.1 for 1 h. The inoculum was then removed; cells were washed with phosphate buffer saline (PBS) and further cultured in complete media. At the indicated time point, lysates were harvested using the Renilla-Glo™ Luciferase Assay System (Promega) as per vendor's protocol. Luciferase signal was detected via luminescence detection using the DTX 880 multimode detector (Beckman Coulter).

### Cell viability assays

Cell viability assays were performed on drug-treated cells using CellTiter-Glo Cell Luminescent Viability Assay (Promega) according to vendor's instructions. This assay measures relative ATP levels. Viability was detected via luminescence detection using the DTX 880 multimode detector (Beckman Coulter) and percent viability was calculated relative to the DMSO control.

### Viral kinetics

Extracellular supernatants were collected from DMSO, sorafenib and siRNA treated samples at various times post-infection. Infectious viral titers were determined by plaque assay on Vero cells (Baer and Kehn-Hall, 2014).

Intracellular RNA was extracted using the RNeasy Mini Kit (Qiagen) according to manufacturer's protocol. Extracellular RNA was extracted from supernatants using the MagMAX™−96 Viral RNA Isolation Kit (Life Technologies) according to manufacturer's protocol. Absolute quantification of RVFV genomic copies was determined by RT-quantitative (q)PCR as previously published (Shafagati et al., 2013).

### Intracellular infectivity assay

To determine intracellular infectious RVFV, 1.0 × 10^6^ HSAECs and Huh7 cells were plated in 6-well plates. Drug treatments and rMP12 infections were performed as described above. Extracellular media supernatants were clarified by centrifugation, while cells were trypsinized and washed twice in complete media. Both media supernatants and cell pellets were stored at −80°C until use. Cell pellets were thawed and resuspended in 500 μl of complete DMEM media. Cells were then lysed by four freeze-thaw cycles and centrifuged at 6000 rpm for 5 min to remove cellular debris. Titers of extra- and intracellular supernatants were determined by plaque assay. After determining the total extra- and intracellular infectivity for the well, the data were plotted as the percent intracellular infectivity of the total (i.e., of both extra- and intracellular supernatants).

### siRNA knockdowns

HSAEC cells, at approximately 70% density in 12-well plates, were transfected with SMARTpool siRNAs targeting B-Raf (10 nM; Dharmacon, #M-003460-03-0005), C-Raf (10 nM; Dharmacon, # M-003601-02-0005), a combination of both (20 nM), or negative control siRNA (10 or 20 nM). All transfections were performed using Dharmafect 1 reagent (Thermo scientific, # T-2001-02). An untreated control with Dharmafect 1 reagent alone was also performed. After 24 h, transfection media was replaced with complete media and cultured for additional 24 h before infection. Protein lysates and extracellular media supernatants were collected at 24 hpi (or 72 h post siRNA transfection). Protein expression was measured by western blot analysis and infectious titers determined by plaque assay.

### Western blots

At 24 h post-infection, cells were collected for western blot analysis. Media was removed, cells washed with PBS, lysed with Blue Lysis Buffer [Mixture of 20 ml T-PER reagent (Thermo Scientific Pierce), 30 ml 2× Tris-glycine SDS sample buffer (Invitrogen), 1.3 ml 1 M DTT, 200 μl 0.5 M EDTA pH 8.0, 80 μl 0.1 M Na_3_VO_3_, 400 μl 0.1 M NaF and 1 complete protease inhibitor cocktail (1 × Halt cocktail, Pierce] and boiled for 10 min. Western blot analysis were performed as previously described (Austin et al., 2012). In brief, membranes were incubated with primary antibodies against B-Raf (1:1000; Abcam # ab117860), C-Raf (1:1000; Abcam # ab124452), or actin, diluted in blocking buffer (PBS containing 3% milk and 0.1% Tween-20) overnight, 4°C. Next day, blots were incubated with appropriate secondary antibodies conjugated to HRP, goat anti-rabbit IgG or goat anti-mouse IgG (1:1000; Thermo Scientific; #32460 (rabbit), # 2430 (mouse) in blocking buffer).

### Animal studies

For sorafenib toxicity experiments, 6–8 week old female BALB/c mice were obtained from Harlan Laboratories. Groups of 3 mice were treated by oral gavage with solvent control (1:4 dilution of 50% ethanol, 50% Kolliphor EL (Sigma-Aldrich, # C5135) in sterile water) or with sorafenib (20 or 40 mg/kg) daily for 10 days. Animals were weighed daily and survival and body condition was monitored for 14 days.

For RVFV infection experiments, 6–8 week old female BALB/c mice were obtained from Harlan Laboratories. Groups of 10 mice were infected with 1 × 10^3^ pfu RVFV ZH501 by sub-cutaneous injection. Mice were pretreated 2 h prior to infection, and each day post infection with 30 mg/kg of sorafenib or solvent control via oral gavage for a total of 10 doses. Body condition was monitored for 14 days post challenge.

All toxicity experiments were carried out in animal bio-safety level 2 (BSL-2) facilities, and ZH501 infection experiments in BSL-3 facilities, in accordance with the National Research Council's Guide for the Care and Use of Laboratory Animals and under GMU IACUC protocols.

### Computational model

A computational model of RVFV infection was developed and analyzed using R Statistical Software (http://www.r-project.org/). Virus titers over time were approximated using the deSolve add-on package and the lsoda function. The aim was to develop the model for the natural infection (without sorafenib) and then use the model to examine potential anti-viral mechanisms associated with sorafenib.

The computational model was a 4-dimentional non-linear system of ordinary differential equations (see Equations 1–4). This model is a simplified version of a computational model developed to study influenza A infection (Handel et al., 2010). This model has four variables, uninfected HSAECs (U), early infected cells which are unable to produce virus (E), virus producing HSAECs (I), and virus produced (V). A schematic of the model is shown in **Figure 6B**. As shown, naïve uninfected HSAECs (U) replicate at some basal rate, *g*, and become infected at rate *b*. Infection immediately converts an uninfected HSAEC into an infected HSAEC, but importantly these newly infected HSAECs are not able to produce virus particles until after some delay, *l*. For this reason, newly infected (but non-virus producing) HSAECs are referred to as early infected (E) and the delay time (i.e., lag) require to convert these cells into infected virus-producing HSAECs (I) is *l*. Thereafter, I produce virus (V) at some rate, *p*, and die at some rate *d*. Finally, newly formed virus can re-infect thereby creating the dynamics seen in this tissue culture infection model.

(1) $$\frac{dU}{dt}\;=gU-bUV$$

(2) $$\frac{dE}{dt}\;=bUV-lE$$

(3) $$\frac{dI}{dt}\;=lE-dI$$

(4) $$\frac{dV}{dt}\;=pI-bUV$$

To estimate initial conditions (which are summarized in Table 1, 5.20 × 10^4^ pfu/ml were added to each well and incubated for 1 h. Thereafter, wells were washed twice and, as discussed above, viral titers in the pooled wash were quantified. Here, there was 1.86 × 10^4^ pfu/ml in the wash, suggesting 3.34 × 10^4^ pfu/ml remained in the well at the start of the infection. In addition, it was estimated that the number of HSAECs in each well at the time of infection was 1.67 × 10^5^ HSAECs/ml. Thus, assuming a well-mixed system (i.e., each HSAEC was infected with a single virus particle), there were 1.34 × 10^5^ uninfected HSAECs (U) and 3.34 × 10^4^ early infected HSAECs (E). For the other two variable initial conditions, we make the assumption that there were zero virus-producing HSAECs (I) at time 0 and because of this there are no viruses present at time 0 [i.e., *V*_(0)_ = 0].

**Table 1:** **Model parameters and initial conditions estimated from data**.

**Symbol**	**Meaning**	**Value**
*g*	Growth rate of uninfected HSAECs	0.742 × 10^−3^ h^−1^
*b*	Infection rate of uninfected HSAECs	0.195 h^−1^ U^−1^
1/*l*	Delay (lag) time from infection to virus production	0.25 h^−1^ (4 h lag)
*d*	Death of virus-producing HSAECs	0.222 × 10^−1^ h^−1^
*p*	Virus production rate	0.531 h^−1^
U(0)	Initial uninfected HSAECs	1.34 × 10^5^ HSAECs ml^−1^
E(0)	Initial early (non-virus producing) HSAECs	3.34 10^4^ HSAECs ml^−1^
I(0)	Initial infected (virus producing) HSAECs	0 HSAECs ml^−1^
V(0)	Initial virus numbers	0 PFU ml^−1^

For model parameter estimates (also summarized in Table 1), the number of HSAECs in a group of control wells were quantified at 0 and 24 h and were determined to be 1.67 × 10^5^ HSAECs/ml and 1.70 × 10^5^ HSAECs/ml, respectively. These data were fit to a simple exponential growth model where the growth rate, *g*, was estimated at 0.742 × 10^−3^/h. The infection rate, *b*, was estimated at 0.195/h/U, which was derived by noting that during the 1 h initial infection in a well with 1.67 × 10^5^ HSAECS, 3.34 × 10^4^ early infected HSAECs (E) were produced [i.e., 3.34 × 10^4^ /(1^*^ 1.67 × 10^5^) = 0.195]. For viral production rate, *p*, the 4–16 h pfu/ml data from **Figure 5A** were fit to a simple exponential growth model where the rate was estimated at 0.531/h. Virus-induced HSAEC death was estimated from data where there was 1.67 × 10^5^ HSAECS/ml at 0 h and 9.77 × 10^4^ HSAECS/ml at 24 h after infection. These data were fit to a simple exponential decay model were *d* was estimated at 2.22 × 10^−2^/h. Finally, the time it takes for an early infected HSAEC (E) to become a virus-producing HSAEC (I) was estimated at 4 h (**Figure 5A**); *l* = 0.25/h.

### Statistics

Statistical significance was determined using Student's unpaired *t*-test to compare the means of test vs. control data. Differences between test and control data were deemed statistically significant if the two-tailed *p* value was ≤ 0.01. For determining CC50 and EC50 values, data was plotted using Prism (GraphPad) using non-linear regression analysis. For the intracellular infectivity assay, Two-Way analysis of variance (ANOVA) was performed using the Holm-Sidak correction for multiple comparisons.

## Results

### FDA-approved drug screening

**Figure 1:**
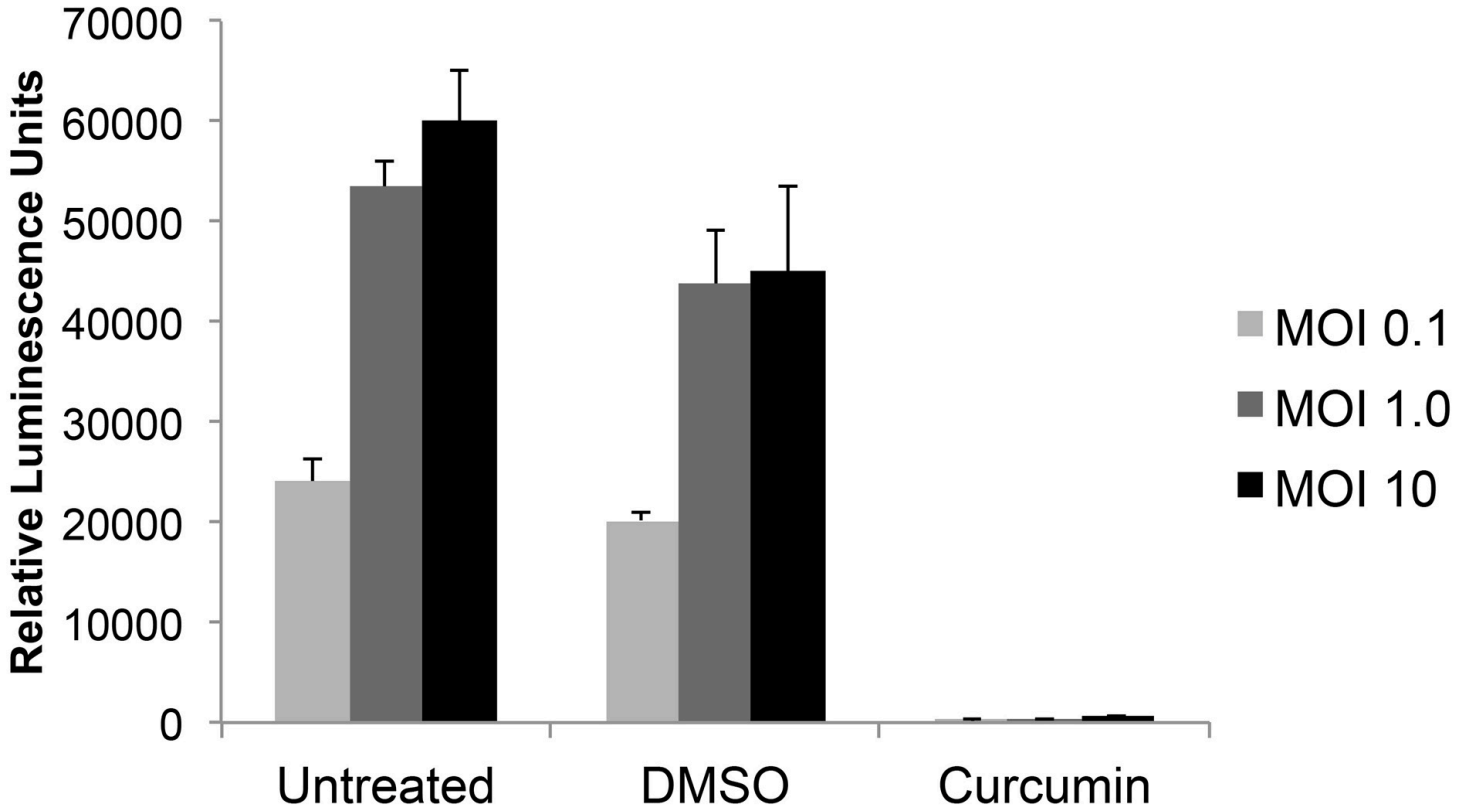
**RVFV luciferase assay development**. Vero cells were either untreated or preincubated with DMSO or 10 μM curcumin for 1 h prior to infection with MP12 ΔNSs-Luc (MOI 0.1, 1.0, or 10). After 1 h infection, virus inoculum was removed, cells washed and media containing respective treatments was added back to cells. Twenty-four hours post infection (hpi), luciferase activity was measured and graphed in relative luminescence units (RLU). The average and standard deviation of three biological replicates are plotted.

In order to determine which of 420 FDA-approved drugs were effective at inhibiting RVFV infection, a high throughput assay was first optimized. The reporter virus, RVFV MP12-Luc, which encodes for *Renilla* luciferase in place of NSs (a non-structural protein that is not essential for virus replication), was utilized to measure virus replication. Vero cells were left untreated or pre-treated with DMSO or curcumin for 1 h prior to infection. Curcumin was used as a positive control for inhibition, since it has been shown to decrease RVFV infection (Narayanan et al., 2012). After pre-treatment, cells were infected with MP12-Luc at a MOI of 0.1, 1.0 or 10. One hour following infection, media containing the respective treatment was added back and cells cultured until 24 h post-infection (hpi). At this time, cells were lysed and relative luminescence determined. In both untreated and DMSO samples, there was an increase in luminescence relative to MOI. As expected, curcumin treatment decreased RVFV infection to levels that were almost undetectable (Figure 1). Z′ factors were calculated for each of the MOIs tested. The Z′ factor is used to assess the quality of an assay in order to predict if it would be useful in a high-throughput setting. A Z′ factor of 1.0 is ideal and cannot be exceeded; between 0.5 and 1.0 denotes an excellent assay; between 0 and 0.5 denotes a marginal assay; and a Z′ factor less than 0 indicates that there is too much overlap between the positive and the negative controls for the assay to be useful (Zhang et al., 1999). Based on our results, the condition chosen for the high-throughput assay was MOI 0.1 since infection at this MOI resulted in the highest Z′ factor (*Z*′ = 0.89).

Using this luciferase reporter assay, a library of FDA-approved drugs was screened. The library comprised of a diverse range of compounds related to oncology, cardiology, inflammation, immunology, neuropsychiatry and analgesia among others. Each drug was used at a concentration of 10 μM to pre-treat Vero cells in a 96-well format. Following pre-treatment the cells were infected with MP12-Luc at a MOI 0.1. Virus inoculum was removed and complete media containing the respective drug was added back to the cells and left on for 24 h. At this time, luciferase reporter activity was measured (Table S1). Compounds that were most efficacious at inhibiting RVFV activity (50% inhibition or greater) were retested in triplicate (Table S2). In addition, cell viability assays were performed to ensure that inhibitory phenotypes were not associated with drug-related toxicity (Table S3). Table 1 lists the compounds that significantly reduced *Renilla* luciferase reporter activity (*p* ≤ 0.01) while maintaining cell viability (≥80%). Some of the classes of drugs that were successful at inhibiting RVFV replication included growth factor receptor inhibitors, microtubule modulators and synthetic estrogen receptor modulators among others. Through, the luciferase reporter assay, it was determined that sorafenib tosylate (sorafenib) was able to reduce RVFV replication with the greatest efficacy, causing a 93% reduction in luciferase luminescence with no toxicity (Table 2).

**Table 2:** **FDA-approved drugs that significantly inhibited MP12 ΔNSs-Luc without reducing cellular viability**.

**Target**	**Drug name**	**Infection vs. DMSO (%)**	***p*-Value**	**Viability (%)**	**References**
Growth factor receptor inhibitors	Sorafenib (Nexavar)	7	0.0001	103	Adnane et al., 2006
	Masitinib (AB1010)	16	0.0002	89	Humbert et al., 2009
	OSI-420 (Desmethyl Erlotinib)	35	0.0006	86	Zerbe et al., 2008
	Pazopanib HCl	39	0.0007	97	Welsh and Fife, 2015
Microtubule assembly and disassembly modulators	Paclitaxel (Taxol)	22	0.0003	85	McGrail et al., 2015
	Vincristine	39	0.0009	87	LaPointe et al., 2013
	Docetaxel (Taxotere)	55	0.0027	108	Mizuuchi et al., 2015
Synthetic estrogen receptor modulators	Toremifene Citrate (Fareston, Acapodene)	28	0.0004	119	Hariri et al., 2015
	Tamoxifen Citrate (Nolvadex)	32	0.0005	102	Vogel, 2015
	Fulvestrant (Faslodex)	57	0.0039	84	Lai et al., 2015
Anti-parasitic	Fenbendazole (Panacur)	19	0.0001	100	Samaee, 2015
	Ivermectin	43	0.0015	95	Arndts et al., 2015
DNA synthesis inhibitor	Gemcitabine HCl (Gemzar)	14	0.0002	81	Sai et al., 2015
	Teniposide (Vumon)	43	0.001	95	Clark and Slevin, 1987
Antifungal agent	Itraconazole (Sporanox)	26	0.0003	88	Feldstein et al., 2015
	Clotrimazole (Canesten)	67	0.0079	94	Chung et al., 2015
Histamine H1 antagonist	Clemastine Fumarate	37	0.0008	119	Apolloni et al., 2014
Calcium antagonist	Manidipine dihydrochloride (CV-4093)	37	0.0008	140	Rizos and Elisaf, 2014
Nucleoside analog	Floxuridine	55	0.0042	108	Vivian and Polli, 2014
Noradrenaline reuptake inhibitor	Maprotiline hydrochloride	59	0.0006	84	Chew and Ong, 2014
Serotonin receptor agonist	Quetiapine fumarate (Seroquel)	59	0.001	81	Pisu et al., 2010

### Sorafenib reduces RVFV replication in multiple cell types

**Figure 2:**
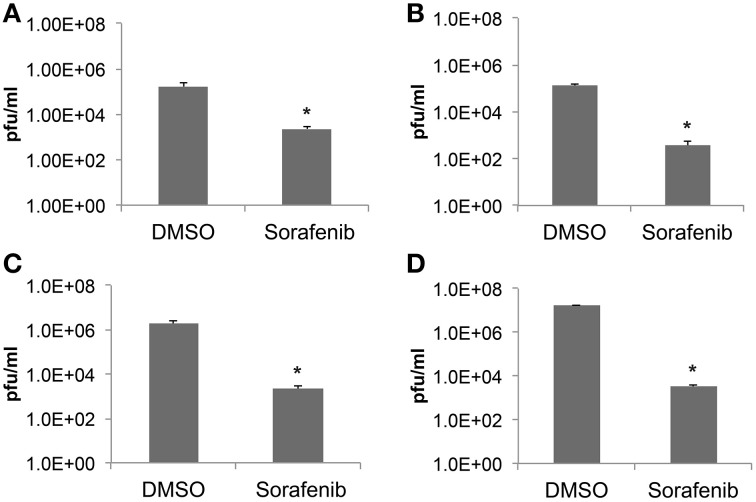
**Sorafenib reduces RVFV replication in multiple cell types**. **(A)** Vero, **(B)** HSAEC, **(C)** Huh7 cells infected with rMP12 (MOI 0.1) or **(D)** Vero cells infected with BSL-3 RVFV strain rZH548 (MOI 0.1) were incubated with either DMSO or sorafenib as described above (Figure 1). Media supernatants were collected 24 hpi and infectious viral titers analyzed by plaque assay. The average and standard deviation of three biological replicates are plotted. ^*^p ≤ 0.01.

To further validate the ability of sorafenib to inhibit RVFV in a variety of cell backgrounds Vero, HSAEC, or Huh7 cells were examined (Figures 2A–C respectively). Cells infected with rMP12 were pre- and post-treated and infectious titers at 24 hpi were determined by plaque assay. The level of infectious rMP12 virus was significantly reduced by approximately 2–3 logs depending on cell type, confirming that the inhibitory effect of sorafenib was not cell type specific. In addition, BSL-3 RVFV strain ZH548 levels were significantly reduced by close to 4 logs, reaffirming the ability of sorafenib to inhibit RVFV *in vitro* (Figure 2D).

### Selectivity index of sorafenib

**Figure 3:**
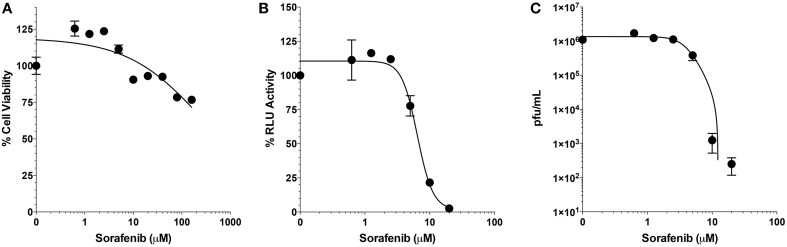
**Selective index of Sorafenib. (A)** Serial 2-fold dilutions (160, 80, 40, 20, 10, 5, 2.5, 1.25, and 0.625 μM) of Sorafenib were used for treatment of Vero cells. After 24 h, cell viability was assessed and plotted as percentage of the control, DMSO alone. Mean and standard deviations are plotted from three biological replicates. Non-linear regression stastics was applied, with the constraint that bottom of the span was to set to less than 10%, to determine the CC_50_. **(B,C)** Serial 2-fold dilutions (20, 10, 5, 2.5, 1.25, and 0.625 μM) of sorafenib were used for treatment of Vero cells infected at a MOI 0.1 with either MP12 ΔNSs-Luc **(B)** or rMP12 **(C)** viruses. After 24 hpi, luciferase activity **(B)** or infectious virus titers **(C)** were measured. For luciferase activity, all treatments concentrations were normalized as a percentage of the control, DMSO alone. For both analyses, the mean and standard deviations from three biological replicates are plotted. Nonlinear regression statistics was applied and the corresponding EC_50_ determined. The EC_50_ of sorafenib for MP12 ΔNSs-Luc and rMP12 were 6.4 and 3.9 μM, respectively.

Selectivity indices (SI) are important measurements in determining the therapeutic potential of an antiviral drug. The SI is defined as the ratio of cytotoxic concentration 50 (CC_50_) to effective concentration 50 (EC_50_) for each compound. To find the CC_50_ for sorafenib, cell viability assays were performed. Two-fold serial dilutions of sorafenib from 160 to 0.625 μM were tested and even at the highest concentration analyzed, viability was not greatly impacted (approximately 76% viability). Concentrations greater than 160 μM were also analyzed, however, these higher concentrations led to sorafenib visibly falling out of solution. Therefore the CC_50_ was determined to be greater than 160 μM (Figure 3A). To determine the EC_50_, two-fold serial dilutions of sorafenib from 20 to 0.625 μM were used to pre- and post-treat Vero cells infected with MP12-Luc (Figure 3B). The percentage of luciferase reporter activity as compared to DMSO control was calculated and the EC_50_ determined to be 6.4 μM. To further validate the EC_50_ measurement, a similar experiment was performed using rMP12 infection in which plaque assays were used to determine the reduction in virus titers (Figure 3C). Within this context the EC_50_ was shown to be 3.9 μM. Using a CC_50_ value of 160 μM, the SI of sorafenib was determined to be >31.74. For comparison purposes, ribavirin has been reported to have a SI of >70 for RVFV MP12 (Furuta et al., 2013).

### Sorafenib affects multiple stages of RVFV lifecycle

**Figure 4:**
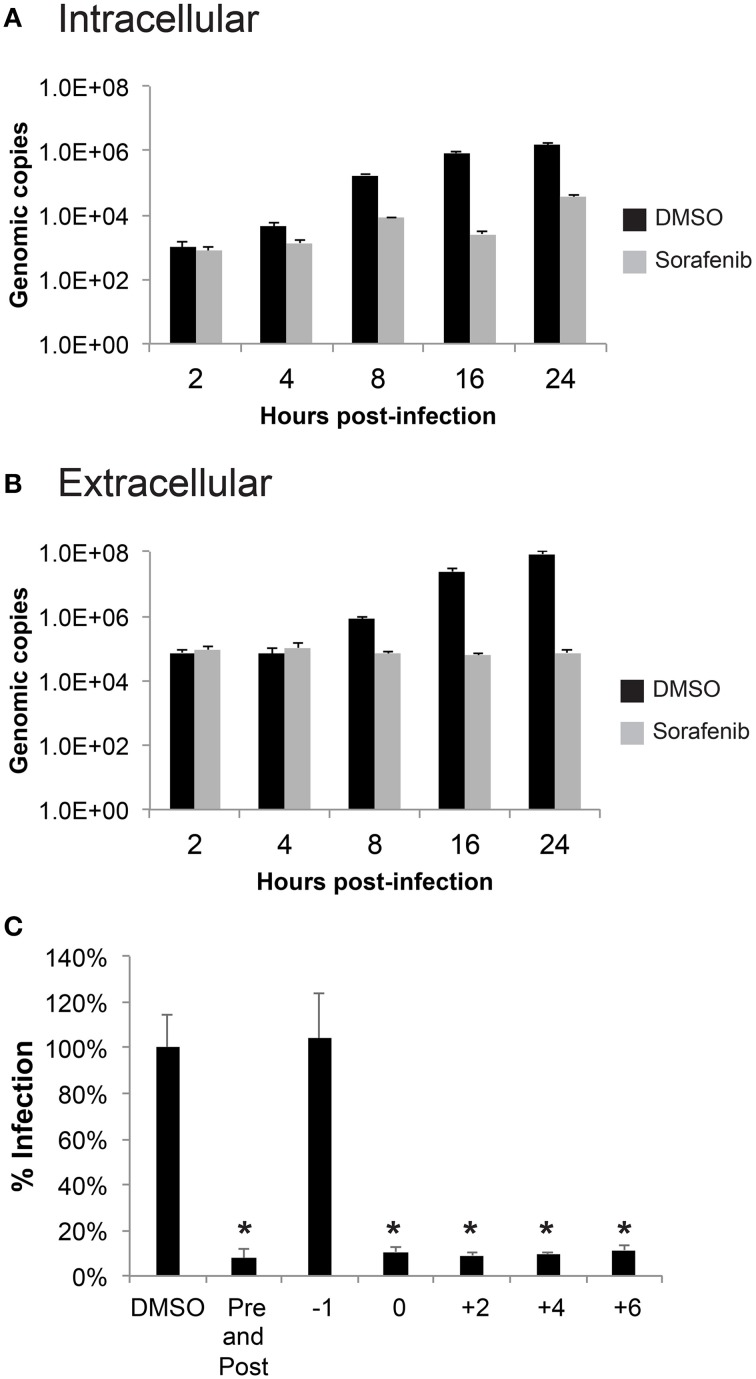
**Sorafenib affects an early stage of infection. (A,B)** HSAECs were pre-treated, infected and post-treated with either DMSO or sorafenib. Supernatants and lysates in RLT buffer were collected at the indicated times post-infection. RNA was extracted from all samples and a qRT-PCR was performed to determine viral genomic copies. The average and standard deviation of three biological replicates are plotted. **(C)** HSAECs were treated at various times relative to MP12 ΔNSs-Luc infection (1 h pre-treatment, at the end of 1 h infection, at 2, 4, 6 hpi, both pre-treated and post-treated). Lysates were collected at 24 hpi and analyzed for luciferase activity. Data is graphed in percentage RLU of the DMSO control. ^*^p ≤ 0.01.

In order to gather more information about which stage of the virus life cycle sorafenib affects, a time course was performed using qRT-PCR to measure accumulation of viral RNA from cell lysates (intracellular RNA, Figure 4A) and supernatants (extracellular RNA, Figure 4B). No difference in viral RNA levels between DMSO and sorafenib treated samples was observed at 2 hpi, suggesting that sorafenib does not impair RVFV entry. A difference in viral RNA levels was detected as early as 4 hpi (Figure 4A). There continued to be only a small increase in intracellular viral RNA levels in sorafenib treated samples over time compared to the exponential increase in DMSO treated samples. This dramatic delay suggests that viral RNA production may be impaired by sorafenib. The extracellular genomic copies in sorafenib treated cells do not significantly change from 0 to 24 h (Figure 4B). The lack of extracellular viral RNA output may simply be a consequence of the decreased of intracellular viral RNA production. However, it is important to note that there was approximately 1.5 log increase in intracellular viral RNA from sorafenib-treated samples observed over time, without a corresponding increase in extracellular RNA levels. These data suggest that an additional virus life cycle step, possibly virus assembly or egress, may be impaired.

Next, a time of addition study was performed where sorafenib was added to cells at various times relative to MP12-Luc infection (Figure 4C). Lysates collected at 24 hpi were analyzed for *Renilla* luciferase reporter activity. No inhibition was noted when cells were pretreated with sorafenib (−1 treatment), supporting the notion that sorafenib does not interfere with viral entry. Furthermore, sorafenib was able to reduce luciferase reporter activity when added to the cells as late as 6 hpi, suggesting that an additional late stage event such as virus assembly or egress could be inhibited.

**Figure 5:**
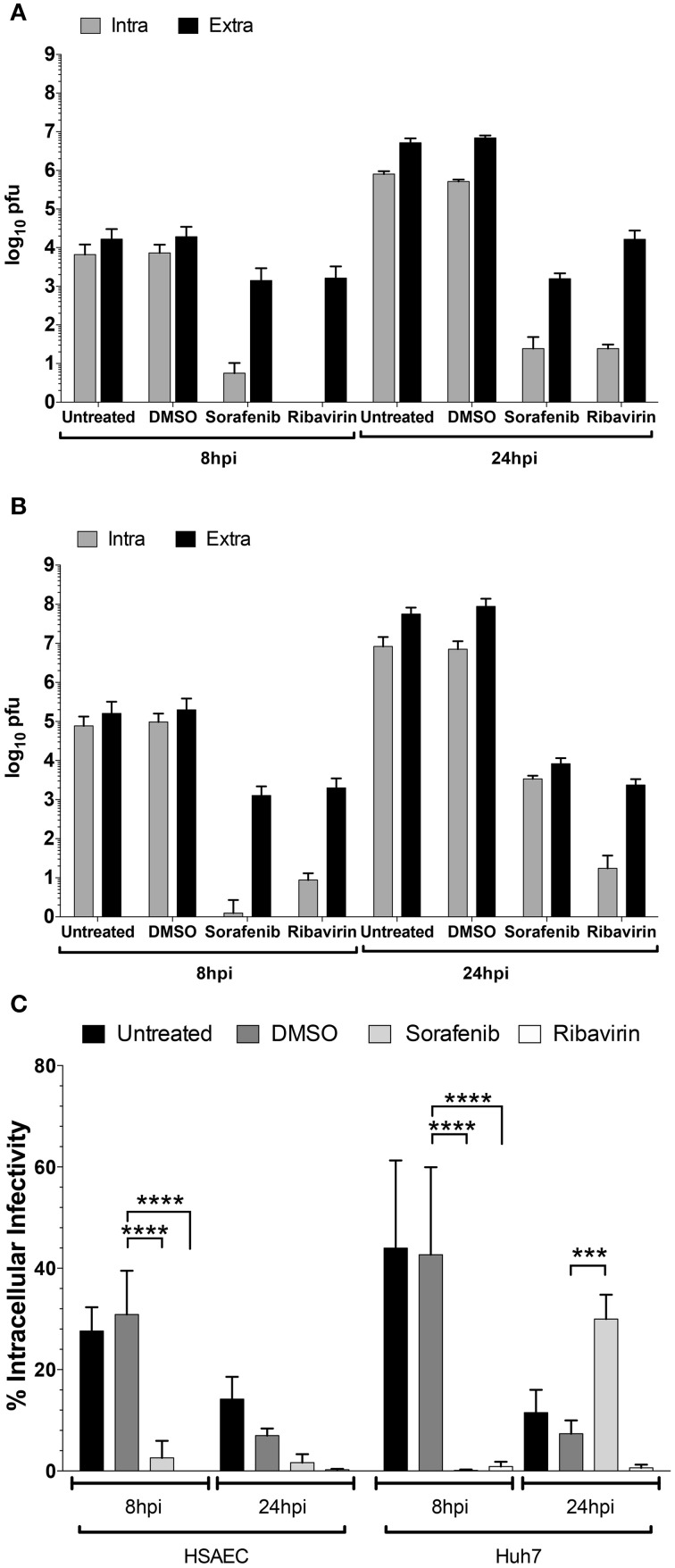
**Increase in rMP12 intracellular infectivity after sorafenib treatment**. **(A)** HSAECs and **(B)** Huh7 cells infected with rMP12 (MOI 0.1) were either left untreated, or treated with DMSO, 10 μM sorafenib, or 82 μM ribavirin as described previously. Intra- and extracellular infectivity per infection as determined by plaque assay is plotted. **(C)** Total infectivity (i.e., sum of intra- and extracellular infectivity) per infection was determined. The percentage of intracellular infectivity relative to the total was plotted for all conditions. Means and standard deviations for four biological replicates are plotted. Two Way ANOVA comparing conditions within each cell background was performed utilizing the Holm-Sidak correction for multiple comparisons. Those comparisons that demonstrated significant differences between DMSO and the other conditions at their respective timepoints are shown. ^***^p ≤ 0.001, ^****^p ≤ 0.0001.

To test whether viral egress was impacted, the amount of intra- and extracellular infectivity was examined at both early (8 hpi) and late (24 hpi) timepoints in both HSAEC and Huh7 cells after rMP12 infection (Figure 5). In addition to sorafenib treatment we also included ribavirin, a general inhibitor of viral RNA dependent RNA polymerases (Arias et al., 2008). Inclusion of ribavirin would allow us to account for inhibitor effects on viral RNA replication alone. In both cell types early in RVFV infection, both sorafenib and ribavirin treatment decreased the amount of intracellular infectivity by several orders of magnitude (Figures 5A,B). This results in a subsequent decrease in detectable virus in media supernatants (i.e., extracellular fraction). At 24 hpi, this pattern starts to alter. In the case of ribavirin the amount of intra- and extracellular virus increases slightly in HSAECs while in Huh7 cells levels remain comparable to the 8 h timepoint. Thus if the block to viral RNA replication alone starts to ease (as in the case of HSAEC cells) virus egress is not impeded. However, after treatment with sorafenib, an increase in the intracellular infectivity was observed especially in Huh7 cells. Comparatively, the percentage of intracellular infectivity (relative to the total infectivity) appears to shift with prolonged sorafenib treatment to a significant degree in Huh7 cells (Figure 5C). Collectively these data implicate two points within the RVFV lifecycle as targets for sorafenib inhibition, replication and egress.

### Computational model of RVFV infection

**Figure 6:**
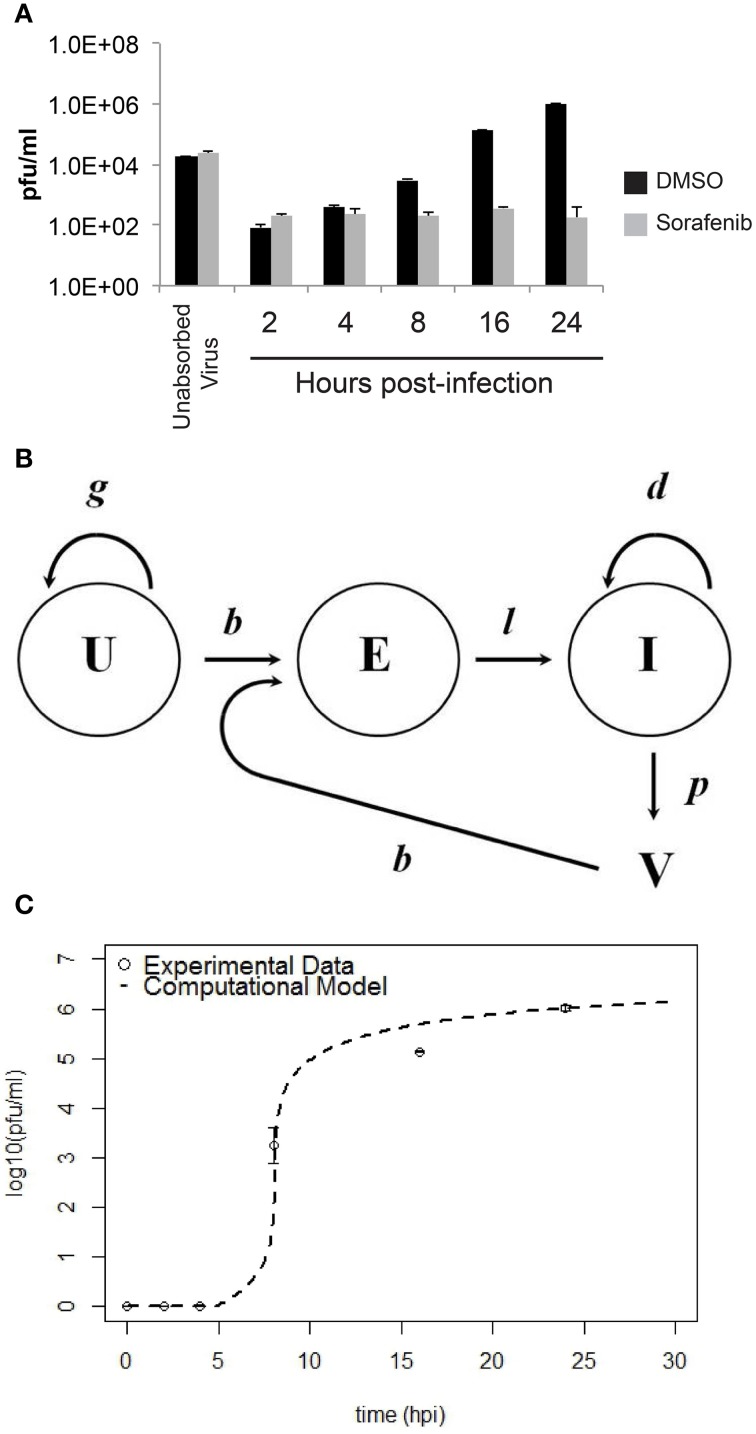
**Comparison between experimental data and the computational model for HSAEC infection. (A)** Virus in the culture supernatant (2–24 hpi) after the addition of sorafenib (gray bars) or DMSO-vehicle (black bars). Data are average pfu/ml ± standard deviations and each bar represents three biological replicates. **(B)** Schematic of the computational model showing uninfected HSAECs (U), early infected HSAECs (E), infected virus-producing HSAECs (I) and Virus particles (V) (see text for additional details). **(C)** Comparison between experimental data and model results. Open circles are the DMSO-vehicle data reproduced from **(A)** and the dashed line is pfu/ml data produced from the computational model using the parameter values summarized in Table 1.

To provide data for a computational model, the growth of RVFV MP12 *in vitro* with and without sorafenib treatment was characterized (summarized in Figure 6A). HSAECs were pre-treated with either DMSO or sorafenib and then infected with RVFV at an MOI of 0.1. After 1 h incubation, the inoculum was removed and the wells were washed with PBS to remove free virus (inoculum and washes are referred to as unabsorbed virus in Figure 6A). Thereafter, the respective drug media (or vehicle control) was put back onto the wells and culture supernatants were collected and viral titers were determined by plaque assay. As shown, sorafenib prevented an increase in viral titers over the entire experimental time course.

A computation model of RVFV infection was developed that included 4 variables and 5 parameters (see Materials and Methods Section for details). A schematic of the model is shown in Figure 6B. Model results obtained using these initial conditions and parameter values (as discussed in Materials and Methods Section) are shown in Figure 6C. As shown, there is excellent agreement between the model and actual pfu/ml data. Indeed, the model predictions at 8, 16, and 24 hpi were 5.75 × 10^1^, 4.85 × 10^5^, and 1.02 × 10^6^ pfu/ml, respectively, and the actual pfu/ml data at these time points (from Figure 6A) were 3.10 × 10^3^, 1.40 × 10^5^, and 1.05 × 10^6^ pfu/ml. To account for biological restrictions, the minimum pfu/ml value was bounded to 1 in the data plotted on a log_10_ scale (Figure 6C). Additionally, 700 pfu/ml was subtracted from all data points prior to plotting Figure 6C to account for background levels of virus detected in the culture supernatant at all time points. These background levels are not related to the infection dynamics, but are experimental artifacts that are primarily the result of (i) virus particles settling on plastic rather than cells that are easily washed off, (ii) particles that are loosely associated with cell surfaces that become dislodged during washing, and (iii) from HSAECs (with internalized viruses) that are actually dislodged during washing. Including this background level of virus has negligible impact on the later time points where the titers are 10^5^ pfu/ml or higher, but in contrast these artifacts can greatly distort data at the early time points where very little *de novo* synthesized virus is expected to be in the culture supernatant.

Because these results suggest the model captured the natural infection dynamics very well, the model was used to begin addressing what step in the virus life cycle could be altered by sorafenib. To this end, pfu/ml over time was modeled after each parameter was manually adjusted. This modeling technique is a type of preliminary sensitivity analysis and begins to test different hypotheses for the drug mechanism of action. As an example, if sorafenib blocks infection, then manually decreasing the infection rate in the model (running the model with a lower *b*) would simulate that mechanism and viral titers in the model output would, in turn, be decreased and match those observed in actual experiments. In contrast, if running the model with a lower *b* had no effect on the model output during the first 24 h, then this observation would support a hypothesis stating the mechanism of action of sorafenib is not at the infection step of the virus life cycle. Table 3 summarizes viral output from the model (pfu/ml at 24 h) when each parameter is adjusted by at least two orders of magnitude. As shown, decreasing HSAECs growth rate (*g*) by 2 orders of magnitude (from 0.742 × 10^−3^ to 0.742 × 10^−5^) had no effect on virus titers at 24 h. Likewise, (i) decreasing the infection rate (*b*) by 2 orders of magnitude, (ii) decreasing the death rate (*d*) by 2 orders of magnitude, or (iii) altering the delay time (1/*l*) had little to no observable effect on viral titers at 24 h. However, in contrast, viral titers at 24 h were dramatically affected by decreasing the virus production rate (*p*). For example, decreasing *p* by an order of magnitude (from 0.531 to 0.0531) resulted in 0 pfu/ml at 24 h. A similar sensitivity-type analysis was done on model initial conditions and results suggest viral titers 24 hpi are not sensitive to changes in variable initial conditions (see Supplemental Data for additional details). Collectively, these observations support a hypothesis where sorafenib prevents virus accumulation in this tissue culture supernatant by primarily acting to prevent viral production within infected HSAECs and/or viral particle release from infected cells. These conclusions are in agreement with the data shown in Figure 4.

**Table 3:** **Model results (pfu/ml at 24 h) with adjusted parameter values**.

**Parameter value**	**pfu/ml at 24 h post infection**
– Actual data (from Figure 6A) –	1.050 × 10^6^
**HSAEC GROWTH RATE (*g*)**	
0.742 × 10^−3^^**^	1.016 × 10^6^
0.742 × 10^−4^	1.014 × 10^6^
0.742 × 10^−5^	1.013 × 10^6^
**INFECTION RATE (*b*)**	
0.195^**^	1.016 × 10^6^
0.0195	1.016 × 10^6^
0.00195	1.015 × 10^6^
**DELAY (LAG) TIME (1/*l*)**	
1 h	1.311 × 10^6^
4 h^**^	1.016 × 10^6^
6 h	8.391 × 10^5^
8 h	6.899 × 10^5^
16 h	3.253 × 10^5^
**DEATH OF HSAECs (*d)***	
0.222	2.295 × 10^5^
0.0222^**^	1.016 × 10^6^
0.00222	1.227 × 10^6^
0.000222	1.251 × 10^6^
**VIRAL PRODUCTION RATE (*p*)**	
0.931	5.278 × 10^5^
0.531^**^	1.016 × 10^6^
0.0531	<1
0.431	7.669 × 10^5^
0.331	5.194 × 10^5^
0.131	3.223 × 10^4^

** *Parameter values that were obtained directly from data. These parameter values are summarized in Table 2 and were used to produce the curve shown in Figure 6C*.

### Loss of raf does not decrease RVFV replication

**Figure 7:**
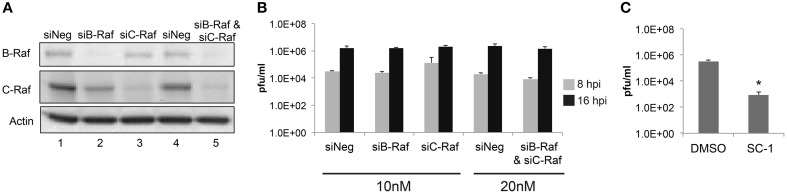
**Loss of Raf does not inhibit RVFV replication**. Targeted siRNA was used to knockdown either B-Raf or C-Raf alone or in combination. **(A)** Western blots show levels of B- and C-Raf proteins. β-Actin was used as a loading control. **(B)** Cells with siRNA knockdown treatment were infected with rMP12 at MOI 3. Supernatants were collected at 8 h and at 16 hpi and plaque assays were performed. **(C)** Cells were pre-treated and post-treated with SC-1 or DMSO and infected with MP12 MOI 0.1. Supernatants were collected 24 hpi and a plaque assay was performed. The average and standard deviation of three biological replicates are plotted. ^*^p ≤ 0.01.

Given that sorafenib was first discovered as a Raf inhibitor, this kinase could be hypothesized to play a major role in the viral lifecycle. To examine the dependency of RVFV infection on Raf function, siRNA knockdowns of both B- and C-Raf were performed followed by infection with MP12. To make sure that the siRNA transfections resulted in a robust knockdown of Raf, western blots probing for both isoforms were performed. B- and C-Raf protein levels were greatly reduced both individually and in combination (Figure 7A). After knockdown, cells were infected with MP12 and infectious viral titers determined at 8 and 16 hpi. A reduction in titers was not observed between siRNA treated cells and control cells (Figure 7B). To further confirm that kinase activity of Raf was not required for RVFV infection, treatment with a sorafenib analog, SC-1, was performed. SC-1 differs from sorafenib in that SC-1 does not affect Raf kinase activity, but retains the ability to inhibit signal transducer and activator of transcription 3 (STAT3) (Yang et al., 2008; Wang et al., 2013). Thus cells treated with SC-1 will retain Raf kinase activity. SC-1 was observed to inhibit RVFV replication between 2 and 3 logs, which was similar to sorafenib inhibition (compare Figure 2 with Figure 7C). These data suggest that Raf kinase activity is not the main anti-viral feature of sorafenib inhibition in RVFV infection.

### *In vivo* effect of sorafenib

**Figure 8:**
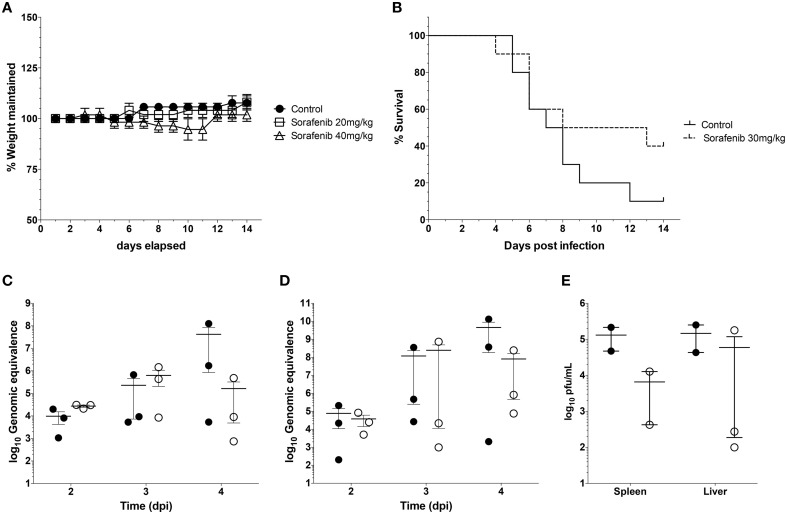
**Sorafenib reduces viremia while increasing survival in RVFV-infected mice**. **(A)** Uninfected BALB/c mice were treated with 20 mg/kg, 40 mg/kg, or solvent control by oral gavage. Animals were monitored daily for weight loss over 14 days. Percentage of weight maintained (relative to starting weight) was determined. Data plotted represents the mean values and standard deviations of three animals per treatment group. **(B)** BALB/c mice were infected with 1 × 10^3^ pfu RVFV ZH501 by sub-cutaneous injection. Mice were pretreated 2 h prior to infection, and each day post infection with 30 mg/kg of sorafenib or solvent control via oral gavage. Animals were monitored for 14 days post challenge and survival curves determined. Data plotted represents 10 animals per treatment group. **(C)** thru **(E)** Mice infected and treated as described above were sacrificed at 2, 3, and 4 days post infection (dpi). Livers and spleens were harvested. RVFV genomic copies within the spleen **(C)** or liver **(D)** were quantified by qRT-PCR for each day. Infectious viral titers **(E)** were determined for day 4 only by plaque assay. Data plotted represents means and standard deviations from three animals per condition. Filled in circles and open circles represent sorafenib and solvent controls respectively.

Finally experiments were performed to examine whether sorafenib exerted a similar influence on viral replication *in vivo* as observed *in vitro*. First, the effect of sorafenib on uninfected BALB/c mice was characterized to determine possible adverse effects by drug treatment. Mice were treated with 20 mg/kg, 40 mg/kg, or solvent control by oral gavage and were monitored daily for weight loss and changes in body condition. The 40 mg/kg dose caused a small decrease in weight between Day 8 and 11 post-treatment, while the 20 mg/kg dose matched the control group weight very closely (Figure 8A). In order to minimize toxicity while still maintaining efficacy of the drug, 30 mg/kg of sorafenib was used to treat mice infected with RVFV ZH501 daily for 10 days. Animals were monitored for 14 days post challenge. Although a trend demonstrating that sorafenib increased survival as compared to control animals was observed, the difference was not statistically significant (Figure 8B). Viral RNA levels in the spleens and livers (Figures 8C,D, respectively) of infected animals were analyzed at 2, 3, and 4 days post-infection (dpi), while day 4 spleen and liver samples were also analyzed for viral titers by plaque assay (Figure 8E). Although not statistically significant, these data demonstrate a trend toward reduction in viral burden at day 4, indicating that sorafenib was effective against RVFV in an *in vivo* model.

## Discussion

The aim of this study was to find a compound that is approved by the FDA for human use and which would be effective against RVFV infection. Repurposing currently approved drugs is desirable as their safety profiles, bioavailability, and mechanisms of action have been extensively studied. Also, in the event of an outbreak, an FDA approved drug may be developed and distributed by a manufacturer that is already producing it with far less delay when compared with a newly discovered compound.

A wide range of compound classes that were effective at inhibiting RVFV were identified. Some compounds however, did have characteristics in common. For example, sorafenib tosylate, masitinib, OSI-420 and pazopanib HCl are all growth factor receptor inhibitors; paclitaxel, vincristine sulfate and docetaxel affect microtubule assembly and disassembly; and toremifene citrate, tamoxifen citrate and fulvestrant are synthetic estrogen receptor modulators. Although none of these drugs are commercially available to treat viral diseases, several of them have been demonstrated to exhibit antiviral activity (Table S1): sorafenib has been shown to improve survival of patients with hepatitis-B virus-related hepatocellular carcinoma (Xu et al., 2015); gemcitabine has potent anti-HIV-2 activity (Beach et al., 2014) as well as anti-influenza A activity (Denisova et al., 2012); paclitaxel inhibits the spread of influenza A virus from cell to cell (Roberts et al., 2015); itraconazole was identified as an effective inhibitor of enterovirus infections (Gao et al., 2015); toremifene has been shown to act as a potent ebola virus (EBOV) inhibitor (Johansen et al., 2013). Tamoxifen exhibited extensive antiviral activities toward herpes simplex virus-1 (Zheng et al., 2014). Pazopanib blocked Andes virus-induced endothelial cell permeability and prevented hantavirus pulmonary syndrome (Gorbunova et al., 2011). Ivermectin was able to reduce Venezuelan equine encephalitis virus replication (Lundberg et al., 2013). All serotypes of dengue virus, as well as West Nile virus were highly sensitive to floxuridine (Fischer et al., 2013).

The drug candidate that proved most effective against RVFV was sorafenib. Sorafenib (Nexavar) is an orally active multi-kinase inhibitor that is FDA-approved for the treatment of hepatocellular carcinoma and renal cell carcinoma. It targets a number of receptor tyrosine kinases, but was primarily identified as a C-Raf inhibitor (Adnane et al., 2006). Sorafenib functions by directly blocking the autophosphorylation of receptor tyrosine kinases (RTKs). These RTKs include VEGFR (vascular endothelial growth factor receptors) 1, 2, and 3, PDGFRβ (platelet derived growth factor receptor), c-Kit and RET which are all pro-angiogenic and are involved in tumorigenesis (Keating and Santoro, 2009). In addition, sorafenib inhibits downstream Raf kinase isoforms including wild-type C-Raf, B-Raf, and the mutant B-Raf V600E. It interacts with Raf kinases by stabilizing the DFG motif (the “Asp-Phe-Gly” motif at the N terminus of the activation loop, Treiber and Shah, 2013) in an inactive conformation (Wilhelm et al., 2006). Furthermore, sorafenib inhibits tumor cell proliferation by targeting the mitogen-activated protein kinase (MAPK) pathway at the level of the Raf kinase (Ravikumar et al., 2011). Overall, sorafenib influences tumor cells and cells of the tumor vasculature.

The potential to repurpose FDA approved drugs has been evaluated for EBOV (Johansen et al., 2013) and a number of biological threat agents including *Bacillus anthracis*, *Francisella tularensis*, *Coxiella burnetii*, and ebola, Marburg, and Lassa fever viruses (Madrid et al., 2013). Selective estrogen receptor modulators (SERMs) were identified as having antiviral effects against EBOV. SERMs were capable of inhibiting EBOV even in cells that were deficient in estrogen receptors, suggesting that these drugs were not acting through their established targets (Johansen et al., 2013). Likewise, the classical mechanism of action of sorafenib appears to be unnecessary for RVFV inhibition. This was demonstrated through siRNA depletion of Raf having little to no effect on viral replication. It should be noted that these results do not definitively exclude Raf from having an influence on RVFV replication, as siRNA depletion experiments do not result in a complete loss of B- or C-Raf protein expression. There is also the possibility that sorafenib could be influencing receptor tyrosine kinase activity such as VEGFR or PDGFR, which was not addressed here. However, SC-1, which is a sorafenib analog lacking kinase-inhibitory activity, decreased RVFV to levels comparable to sorafenib. These results support the hypothesis that non-classical targets of sorafenib are important for RVFV replication.

Recently, signal transducer and activator of transcription 3 (STAT3) was identified as an additional target of sorafenib (Yang et al., 2008). Phosphorylation of STAT3 was inhibited following sorafenib treatment, which was associated with inhibition of cell proliferation and induction of apoptosis in medulloblastomas. Both sorafenib and SC-1 operate by preventing STAT3 phosphorylation and thus preventing its subsequent activation. STAT3 phosphorylation is inhibited by directly activating the upstream protein tyrosine phosphatase Src homology 2-domain containing tyrosine phosphatase 1 (SHP-1) (Liu et al., 2013). Therefore, proteins downstream of SHP-1 may be influencing RVFV replication. SHP-1 affects a vast number of proteins apart from STAT3. These include Akt, NF-κB, ERK, and JNK (Chong and Maiese, 2007). As a result, unearthing a single cellular target of sorafenib that RVFV is exploiting to propagate its life cycle might prove to be challenging.

Sorafenib was effective at inhibiting RVFV *in vitro* with an SI of >31, but only demonstrated limited efficacy in a subcutaneous mouse model of RVFV infection. There are a number of possibilities that could explain the limited improvement in animal survival observed. The half-life of sorafenib is quite long (25–48 h) (Flaherty et al., 2008), thus a once a day dosing regimen was chosen. Altering the dosing schedule and dosage amounts could provide for improvement in animal survival. However, the most significant contributing factor is that the target for sorafenib inhibition in RVFV infected cells is non-canonical (as discussed above). While sorafenib has potent activity against Raf and receptor tyrosine kinases, the potency against the as yet to be determined cellular target during RVFV infection is unclear. Sorafenib target identification in the context of RVFV infection is necessary to allow further refinement of sorafenib and/or selection of additional lead candidates for drug development efforts.

Mechanism of action and computational modeling studies indicated that sorafenib influences at least two steps in the viral infectious cycle, RNA synthesis and virus assembly/egress. The degree to which viral egress is impacted appears to be cell type dependent as shown when comparing % intracellular infectivity between HSAEC vs. Huh7 cells (Figure 5C). Whether, this is due to differing levels of the protein targeted by sorafenib inhibition in Huh7 cells or possible dual roles of this cellular factor in both virus assembly and egress remains to be determined. Recently Descamps et al. demonstrated that multiple steps of hepatitis C virus infection were inhibited by sorafenib treatment, namely entry and production of infectious viral particles (Descamps et al., 2015). RVFV entry has been shown to be largely caveola-mediated (Harmon et al., 2012). While caveola-mediated endocytosis is a complex process regulated in part by tyrosine kinases and phosphatases, RVFV dependence on these signaling cascades is not entirely known. Thus, as sorafenib targets receptor tyrosine kinases, the possibility of inhibiting viral entry is not unexpected. However pre-treatment of cells with sorafenib had no effect on RVFV replication and computational modeling supports the idea that sorafenib has no impact on RVFV entry. Furthermore, the siRNA experiments and SC-1 treatment suggest that sorafenib likely acts independent of its tyrosine kinase inhibitory activity. RVFV RNA synthesis was dramatically altered following sorafenib treatment. However, these studies did not differentiate between viral transcription or RNA replication as the qRT-PCR assay utilized does not discriminate between RNA species. Additional studies are necessary to determine if sorafenib is influencing primary mRNA transcription, replication, or secondary mRNA transcription. A number of cell based systems have been developed to differentiate between primary mRNA transcription and replication (Habjan et al., 2008; Klemm et al., 2013) which will be useful for this analysis.

The computational model developed herein provided additional evidence that sorafenib influences RVFV post entry and results in a decrease in infectious virus released. The model was fully parameterized using actual experimental data and appeared to capture the infection dynamics quite well. Results for our *in vitro* infection model suggested that HSAECs were infected quickly following the addition of virus, but new viruses were not released in the extracellular media until approximately 8 hpi. At that time, all HSAECs have become infected and shortly thereafter all infected HSAECs are assembling new virus. When the model was used to simulate and assess potential anti-viral mechanisms associated with sorafenib, results suggested that the inhibitor had no effect on virus uptake by HSAECs *per se*. Rather, sorafenib was acting on infected HSAECs by either inhibiting RNA production, preventing formation of mature intracellular particles, or preventing their release. These conclusions agreed well with additional data that showed sorafenib could exert its effect when added up to 6 h post the start of infection, increased intracellular infectivity (especially in Huh7 cells) at 24 hpi and had no effect on viral RNA accumulation during the first 4 h of infection. Future studies will focus on refinement of this model to include additional steps of the virus lifecycle cycle to allow a more detailed analysis to be performed and narrow down the mechanism of sorafenib inhibition.

## Author contributions

AB, KK, CD, BG, IH, SS and AN designed experiments. AB, NB, CD, SS, LL and IH performed the experiments. AB, KK, CD, BG, NB and AN wrote and edited the manuscript.

## Funding

This material is based upon work supported by the U.S. Department of Homeland Security under Cooperative Agreement Number DHS 2010-ST-061-AG0002. The views and conclusions contained in this document are those of the authors and should not be interpreted as necessarily representing the official polices, either expressed or implied, of the U.S. Department of Homeland Security. Publication of this article was funded in part by the George Mason University Libraries Open Access Publishing Fund.

### Conflict of interest statement

The authors declare that the research was conducted in the absence of any commercial or financial relationships that could be construed as a potential conflict of interest.
