# Supplementary material for: Repurposing FDA-approved drugs as therapeutics to treat Rift Valley fever virus infection
Source: Front Microbiol. 2015 Jul 8;6:676. doi: 10.3389/fmicb.2015.00676 (PMC4495339; doi:10.3389/fmicb.2015.00676)
Supplement: Supplementary file 4 [file Presentation1.PDF]

Table S4.

Drug	Affected viruses	Reference(s)
Sorafenib	Hepatitis-B virus-related hepatocellular carcinoma	(Xu et al. 2015)
	Hepatitis-C virus-related hepatocellular carcinoma	(Descamps et al. 2015)
Gemcitabine	HIV-2	(Beach et al. 2014)
	Influenza A	(Denisova et al. 2012)
Paclitaxel	Influenza A	(Roberts, Manicassamy, and Lamb 2015)
Itraconazole	Enterovirus	(Gao et al. 2015)
Toremifene	Ebola virus	(Johansen et al. 2013)
Tamoxifen	Herpes Simplex Virus-1	(Zheng et al. 2014)
Pazopanib	Andes virus	(Gorbunova et al. 2011)
	Hantavirus	
Ivermectin	Venezuelan Equine Encephalitis Virus	(Lundberg et al. 2013)
Floxuridine	All serotypes of Dengue virus	(Fischer et al. 2013)
	West Nile virus	

Additional Model Results. As discussed in the main text, preliminary analysis of the model suggested lowering the infection rate, *b,* to simulate a potential anti-viral mechanism of sorafenib did not reduce viral titers (PFU/ml) at 24 hours to levels observed in actual experiments. This suggested sorafenib's main anti-viral mechanism may not be inhibiting infection. However, it is noted that an initial condition for the model was that Early Infected HSAEC (E) = 3.34 x 10^4 HSAECs ml^{-1} (see Fig. S1). And, if sorafenib, which was added to the culture 2 hours prior to addition of the virus, blocked the infection stage of the life cycle (i.e., decrease *b*), then the model may not be able to simulate that specific anti-viral mechanism because E is produced at a rate *b.* In other words, there will still be viral dynamics in the model even if *b* is set to 0 because of the initial condition on E, in which case the system shown in eqs. 1-4 become a linear system and V(t) = *p*(I). Fig. S2 shows the viral dynamics, V(t), when *b* is set to 0 and all other parameters and initial conditions remain unchanged (summarized in Table 1). As shown, virus increased rapidly with little effect on viral titers at 24 hpi.

A first step in addressing this was to run the model after changing the initial conditions such that E(0) = 0 and V(0) = 3.34 x 10^4 while keeping the other two initial conditions 0. In doing so, all model dynamics now involve *b.* This is a reasonable modification from an experimental view point because it is assumed that if infection was blocked by sorafenib then all virus particles added to the culture would remain extracellular, unable to be taken up by U [note: V(t) in this model is virus particles (PFU/ml) in the culture supernatant]. Furthermore, the data suggests that since there is no difference between the number of virus particles in the culture at time 0 in natural infections (no sorafenib) compared with sorafenib infections (see Fig. 6A), virus particles in the sorafenib culture will be suspended in the culture and/or sitting on the HSAEC cell surface unable to be gain entry and infect. As shown in Fig. S3, there is little effect on PFU/ml at the later time points (8, 16, 24 hpi, compared to Fig. 6C). Likewise, there is little effect on PFU/ml when the infection rate is decreased by 3 orders of magnitude (Fig. S4). Collectively, these results suggest V(t) at later time points is not sensitive to changes in the infection rate *b* or to changes in the initial conditions for V or E, and that V(t) in this infection is only sensitive to changes in *p,* the virus production rate. The results cannot rule out that sorafenib may be acting to block infection, but if so, this mechanism alone cannot explain the observed dynamics and that the primary effect of the drug affecting viral production.

As a final analysis, all variables were plotted on the same graph for the natural infection (no sorafenib) and with sorafenib (simulated by setting p = 0.0531); the results are shown if Fig. S5 and Fig. S6, respectively. As shown for the natural infection, model dynamics change significantly at around 8 hours. At this time point, virus titers (V) increase drastically and the number of virus producing HSAECs (I) outnumber the number of uninfected (U) and early infected (E) HSAECs. These observations suggest the addition of sorafenib after approximately 8 hours post infection would have limited effect on viral titers and, furthermore, allow the possibility that sorafenib could be added to the culture between 0-8 hours post infection and still show an anti-viral effect. Here, it is noted that based on viral PCR data suggested sorafenib could still produce and anti-viral effect when added 6 hours post infection (Fig. 4C). In the presence of sorafenib, approximately 4 hours post infection appears to be a critical time point in the infection dynamics (Fig. S6). As shown, at approximately 4 hours post infection the quantity

of latently infected HSAECs (I) outnumber the quantity of early infected HSAECs (E), and virus production is 0 hr^{-1} at 4 hours and remains zero through 24 hours. A reasonable biological interpretation of this is that virus particles introduced at the start of the infection are absorbed by HSAECs with kinetics similar to those observed without drug, and these early infected HSAECs progress to latently infected HSAECs (I) by 4 hours; however, the latently infected HSAECs are unable to release virus particles into the supernatant. These model results agree well with data shown in Fig. 4, where it was shown that in the presence of sorafenib: (i) viral RNA copies inside HSAECs increased identically to that observed in non-drug infection values during the first 4 hours post-infection, (ii) at which time the intracellular RNA increase stalled, and (iii) thereafter, there was a lack of viral RNA detected in the culture supernatant.

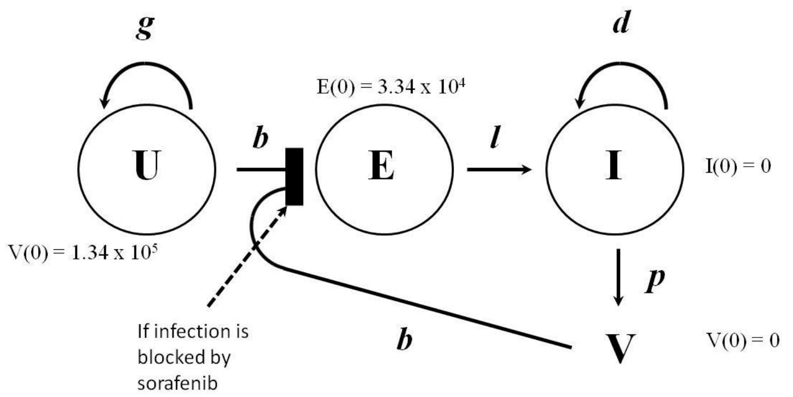

Fig. S1. Modified schematic of model showing initial conditions and model dynamics if infection (*b*) is blocked by sorafenib.

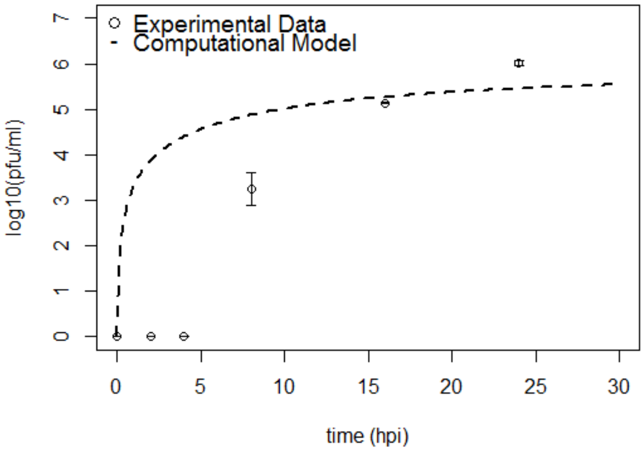

Fig. S2. Viral dynamics when *b* is set to 0. The infection rate, *b*, was set to 0 and all other parameters and initial conditions were left as summarized in Table 2.

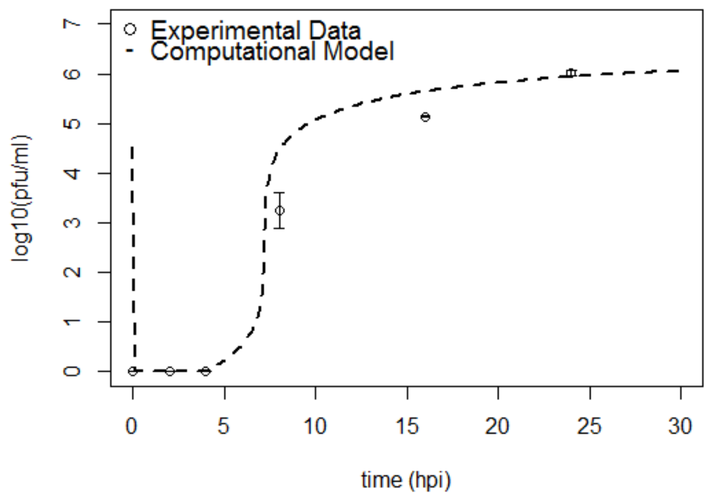

Fig. S3. Viral dynamics when E(0) = 0 and V(0) = 3.34 x 10^4. The initial conditions for E and V were adjusted and the other two initial conditions were left at 0. All parameters were left unchanged (as summarized in Table 2).

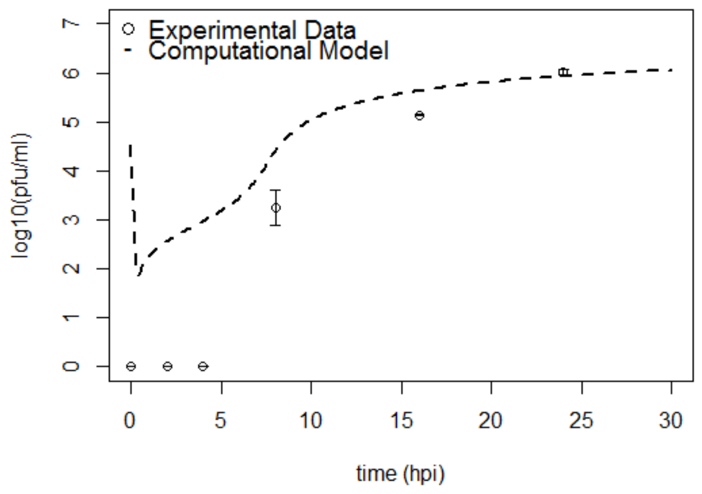

Fig. S4. Viral dynamics when E(0) = 0, V(0) = 3.34 x 10^4 and *b* =0 .000195. The initial conditions for E and V were adjusted and the other two initial conditions were left at 0. The infection rate was decreased by 3 orders of magnitude (*b* = 0.000195) and all parameters were left unchanged (as summarized in Table 2).

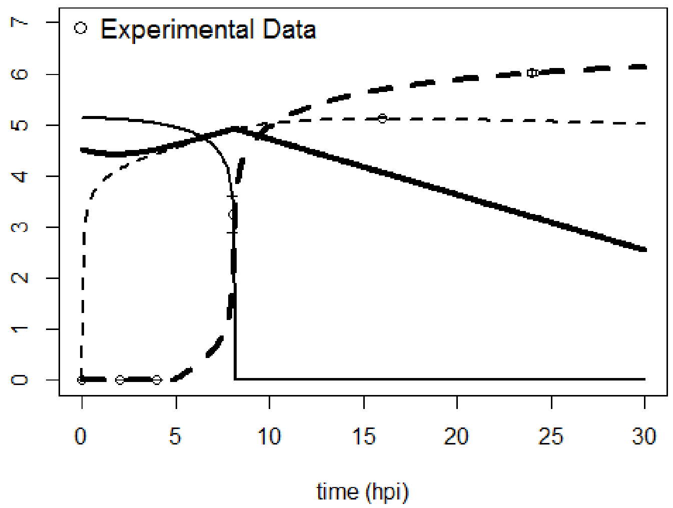

Fig. S5. U(t), E(t), I(t) and V(t) for the natural (no sorafenib) infection. This plot shows numerical approximations for all variables and was generated using the initial conditions and parameter values summarized in Table 2. [U(t) = thin solid line: E(t) = thick solid line: I(t) = thin dashed line: V(t) = thick dashed line]

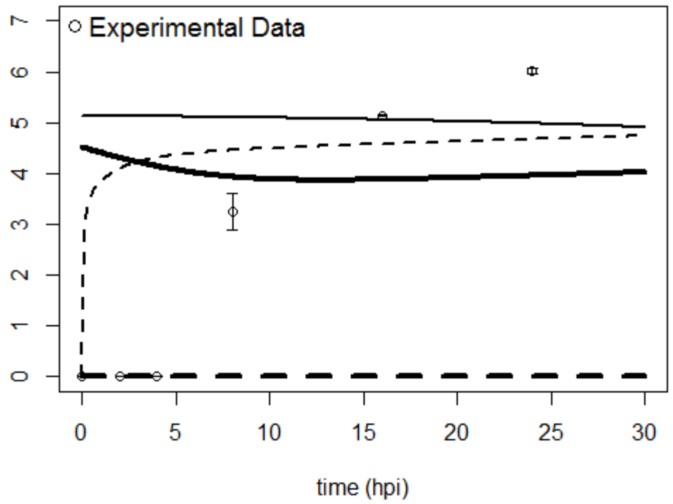

Fig. S6. U(t), E(t), I(t) and V(t) for the natural (no sorafenib) infection. This plot shows numerical approximations for all variables and was generated using the initial conditions and parameter values summarized in Table 2 except p=0.0531. [U(t) = thin solid line: E(t) = thick solid line: I(t) = thin dashed line: V(t) = thick dashed line]

References:

Beach, Lauren B., Jonathan M. Rawson, Baek Kim, Steven E. Patterson, and Louis M. Mansky. 2014. "Novel Inhibitors of Human Immunodeficiency Virus Type 2 Infectivity." *The Journal of General Virology* 95 (Pt 12): 2778–83. doi:10.1099/vir.0.069864-0.

Denisova, Oxana V., Laura Kakkola, Lin Feng, Jakob Stenman, Ashwini Nagaraj, Johanna Lampe, Bhagwan Yadav, et al. 2012. "Obatoclax, Saliphenylhalamide, and Gemcitabine Inhibit Influenza a Virus Infection." *The Journal of Biological Chemistry* 287 (42): 35324–32. doi:10.1074/jbc.M112.392142.

Descamps, Véronique, François Helle, Christophe Louandre, Elodie Martin, Etienne Brochot, Laure Izquierdo, Carole Fournier, et al. 2015. "The Kinase-Inhibitor Sorafenib Inhibits Multiple Steps of the Hepatitis C Virus Infectious Cycle in Vitro." *Antiviral Research*. Accessed April 3. doi:10.1016/j.antiviral.2015.03.012.

Fischer, Matthew A., Jessica L. Smith, David Shum, David A. Stein, Christopher Parkins, Bhavneet Bhinder, Constantin Radu, et al. 2013. "Flaviviruses Are Sensitive to Inhibition of Thymidine Synthesis Pathways." *Journal of Virology* 87 (17): 9411–19. doi:10.1128/JVI.00101-13.

Gao, Qianqian, Shilin Yuan, Chao Zhang, Ying Wang, Yizhuo Wang, Guimei He, Shuyi Zhang, Ralf Altmeyer, and Gang Zou. 2015. "Discovery of Itraconazole with Broad-Spectrum In Vitro Anti-Enterovirus Activity That Targets Nonstructural Protein 3A." *Antimicrobial Agents and Chemotherapy*, February. doi:10.1128/AAC.05108-14.

Gorbunova, Elena E., Irina N. Gavrilovskaya, Timothy Pepini, and Erich R. Mackow. 2011. "VEGFR2 and Src Kinase Inhibitors Suppress Andes Virus-Induced Endothelial Cell Permeability." *Journal of Virology* 85 (5): 2296–2303. doi:10.1128/JVI.02319-10.

Johansen, Lisa M., Jennifer M. Brannan, Sue E. Delos, Charles J. Shoemaker, Andrea Stossel, Calli Lear, Benjamin G. Hoffstrom, et al. 2013. "FDA-Approved Selective Estrogen Receptor Modulators Inhibit Ebola Virus Infection." *Science Translational Medicine* 5 (190): 190ra79. doi:10.1126/scitranslmed.3005471.

Lundberg, Lindsay, Chelsea Pinkham, Alan Baer, Moushimi Amaya, Aarthi Narayanan, Kylie M. Wagstaff, David A. Jans, and Kylene Kehn-Hall. 2013. "Nuclear Import and Export Inhibitors Alter Capsid Protein Distribution in Mammalian Cells and Reduce Venezuelan Equine Encephalitis Virus Replication." *Antiviral Research* 100 (3): 662–72. doi:10.1016/j.antiviral.2013.10.004.

Roberts, Kari L., Balaji Manicassamy, and Robert A. Lamb. 2015. "Influenza A Virus Uses Intercellular Connections to Spread to Neighboring Cells." *Journal of Virology* 89 (3): 1537–49. doi:10.1128/JVI.03306-14.

Xu, Li, Hengjun Gao, Junting Huang, Haoyuan Wang, Zhongguo Zhou, Yaojun Zhang, Shaohua Li, and Minshan Chen. 2015. "Antiviral Therapy Improves Survival of Patients with Hepatitis B Virus-Related Hepatocellular Carcinoma Who Treated with Sorafenib." *Journal of Gastroenterology and Hepatology*, January. doi:10.1111/jgh.12910.

Zheng, Kai, Maoyun Chen, Yangfei Xiang, Kaiqi Ma, Fujun Jin, Xiao Wang, Xiaoyan Wang, Shaoxiang Wang, and Yifei Wang. 2014. "Inhibition of Herpes Simplex Virus Type 1 Entry by Chloride Channel Inhibitors Tamoxifen and NPPB." *Biochemical and Biophysical Research Communications* 446 (4): 990–96. doi:10.1016/j.bbrc.2014.03.050.
